# Stress can lead to an increase in smartphone use in the context of texting while walking

**DOI:** 10.3389/fpsyg.2022.760107

**Published:** 2022-09-08

**Authors:** Maria Lilian Alcaraz, Élise Labonté-LeMoyne, Sonia Lupien, Sylvain Sénécal, Ann-Frances Cameron, François Bellavance, Pierre-Majorique Léger

**Affiliations:** ^1^HEC Montréal, Montreal, QC, Canada; ^2^Centre for Studies on Human Stress, Montreal Mental Health University Institute, Montreal, QC, Canada; ^3^Department of Psychiatry, Faculty of Medicine, University of Montreal, Montreal, QC, Canada

**Keywords:** stress, texting and walking, smartphone usage, physiological stress biomarkers, texting, psychological stress

## Abstract

Texting while walking (TWW) is a dangerous behavior that can lead to injury and even death. While several studies have examined the relationship between smartphone use and stress, to our knowledge no studies have yet investigated the relationship between stress and TWW. The objective of the present study was to investigate this relationship by examining the effects of stress on TWW, the effects of TWW on subsequent stress, and the effect of stress on multitasking performance. A total of 80 participants completed two sequential tasks in a laboratory while they walked on a treadmill and responded to a biological motion stimulus imitating the movement of another pedestrian. In the unrestricted task, participants were given the choice to use their personal phones. In the controlled task, they carried a text conversation with a research assistant while they walked and responded to the stimulus. Stress was measured *via* questionnaire and saliva collection for measure of cortisol (a stress hormone) before and after each task. Results show that greater psychological stress and cortisol variations were associated with a greater number of phone uses during the unrestricted task. Greater phone use during the unrestricted task was associated with lower subsequent psychological stress in women and total time of phone use was correlated with subsequent cortisol levels. Stress measured before the controlled task had no effect on multitasking performance, but participants with moderate performance were those with the highest cortisol levels. Our results suggest that stress could be a precursor to TWW and that it could affect a pedestrian’s ability to stay safe when using their smartphone.

## Introduction

Smartphones have become an integral part of daily life and their adoption continues to grow. It is predicted that by 2026, 7.5 billion people will own smartphones across the globe (Statista, 2019). Yet smartphones are way more than traditional telecommunication devices: they allow us to access the Internet and use online banking services, on-demand entertainment, social media, and much more, anywhere and at any time. Given this versatility, it is unsurprising that smartphones encourage users to spend more time using their phones while performing other things (i.e., multitasking; Soukup, 2015). One such type of multitasking is texting while walking (TWW).

TWW is a dangerous behavior that involves both a lack of attention to the environment and a slower walking pace, which puts individuals at a greater risk of collisions and injuries (Parr et al., 2014). One study reported that pedestrians who texted while walking took 18% longer to cross an intersection and performed at least one risky behavior on average compared to non-distracted pedestrians (Thompson et al., 2013). Other studies have shown that pedestrians who use their smartphones while walking are more likely to be hit by moving vehicles and suffer from other types of accidents (Schwebel et al., 2012; Byington and Schwebel, 2013; Kim et al., 2017). Moreover, as smartphone usage continues to increase, accidents related to its use in pedestrians are also on the rise (Nasar and Troyer, 2013). In 2015, a study conducted on five of the busiest intersections in Manhattan showed that a third of pedestrians crossing the street on a green light and half of pedestrians crossing on a red light were distracted by an electronic device (Basch et al., 2015). A more recent study published in 2019 show that 20% of pedestrians used their smartphone while crossing the street (Horberry et al., 2019). Interestingly, a recent PEW research center survey found that 75% of adults found it acceptable to use their phone while walking down the street (Adams et al., 2020).

As stress can affect the cognitive abilities necessary to texting while walking and has been found to be both a predictor (Boumosleh and Jaalouk, 2017; Cho et al., 2017; Han et al., 2017; Gökçearslan et al., 2018) and a consequence of (Chesley, 2005; Lepp et al., 2013; Murdock, 2013; Lee et al., 2014; Hawi and Samaha, 2017) smartphone use, this research project aimed to evaluate the combination of stress and TWW.

### Measuring physiological stress

When a situation is interpreted as being stressful, the hypothalamic–pituitary–adrenal (HPA) axis is activated and neurons in the hypothalamus, a structure located in the brain and named the “master gland,” releases a hormone called corticotropin-releasing hormone (CRH). The release of CRH triggers subsequent secretion and release of another hormone called adrenocorticotropin (ACTH) from the pituitary gland, also located in the brain. When ACTH is secreted by the pituitary gland, it travels in the blood flow and reaches the adrenal glands, which are located above the kidneys, and triggers secretion of the so-called stress hormones. There are two main stress hormones, the glucocorticoids (called cortisol in humans), and the catecholamines (adrenaline and noradrenaline; Kirschbaum et al., 1993). Salivary cortisol has become a favored measure of the acute human stress response in experimental research as it is more easily obtained than other measures such as blood or urine tests (Kirschbaum et al., 1993). Salivary cortisol is measured from small saliva samples, having research participants spit into a small tube, and can be repeated as often as desired to measure changes in stress in response to an experimental situation (Kirschbaum et al., 1993). A stress response can be observed in the measurement of cortisol within 15 min (Campbell and Ehlert, 2011). Because physiological and psychological stress have been shown to not always covary (Hjortskov et al., 2004; Campbell and Ehlert, 2011; Ali et al., 2017), it is possible that the effects of smartphone use on psychological and physiological stress are distinct.

### Stress and smartphone use

Although most of the literature has focused on problematic smartphone use (i.e., smartphone use with the characteristics of addiction; Bosch et al., 2015; Elhai et al., 2016; Taylor et al., 2020; Yang et al., 2021; Wickord and Quaiser-Pohl, 2022), even non-problematic smartphone use has been identified as both a consequence and a predictor of stress (Igarashi et al., 2005; Jin and Peña, 2010; Lepp et al., 2013). Different explanations have been proposed to explain the mechanism behind the stress and smartphone use relationship. Some suggest that in the case of problematic smartphone use, it is a way to cope with already-existing psychopathology (e.g., anxiety, depression; Billieux, 2012). A similar explanation has been proposed for non-problematic smartphone use, the “social blanket phenomenon,” wherein one’s smartphone may function as a coping tool against acute social stressors (Hunter et al., 2018). In cases of either problematic and non-problematic smartphone use, it is possible that an over-reliance on smartphones for reassurance-seeking purposes is also a feature and a maintenance factor of stress and anxiety disorders (Cougle et al., 2011; Nikmanesh et al., 2014).

To our knowledge, no studies have examined the acute effects of smartphone use on psychological and physiological stress. In a study by Hunter et al. (2018), the mere presence of the participants’ smartphones helped reduce their perceived social exclusion as well as the concentration of salivary alpha amylase (a hormone associated with adrenergic activation) following the stressful event. However, no studies have examined whether actual smartphone use, rather than its mere presence, also has this effect. Moreover, no studies have reported on the effect of smartphone use on subsequent physiological stress. A review on technology’s effect on physiological stress concluded that human interaction with computers can result in increased physiological stress markers (Riedl, 2012), but it is unclear whether this could also apply to smartphones.

### Stress and cognition

Task-switching paradigms are a useful way of studying the cognitive processes involved in TWW, a dual-task situation where pedestrians are attending to their device while also attempting to pay attention to their surroundings. In task-switching experiments, participants “switch” between different tasks which require attention to a particular stimulus (Kiesel et al., 2010). As stress and associated stress hormones are well-known modulator of high-level cognitive tasks (Courtemanche et al., 2019), it is likely that they may have an impact on cognition during TWW. However, the evidence on the effects of stress on high cognitive function is mixed. While stress has been shown to hinder performance in tasks of cognitive flexibility (Kalia et al., 2018) and on working memory (Oei et al., 2006), it has also been reported to facilitate task-switching (Steinhauser et al., 2007; Lin et al., 2018), increase performance in examinations (Kofman et al., 2006), and enhance working memory in certain conditions (Yuen et al., 2009; Schoofs et al., 2013). The underlying mechanisms for the effects of stress on cognition remain unclear. Some suggest that stress may impair higher-order cognitive faculties in order to facilitate well-learned and habitual behavior, which allows for faster responses (Gagnon and Wagner, 2016; Vogel et al., 2016). However, others propose that stress may help to easily relocate cognitive resources to stimuli that are relevant to the stressor (LeBlanc, 2009; Mather and Sutherland, 2011; Plessow et al., 2011). One of the first attempts to describe the relationship between stress and cognition was Yerkes and Dodson’s (Yerkes and Dodson, 1908) inverted U law, which posits that performance in cognitive tasks is best at an optimal stress level, and that too-high or too-low stress can be detrimental to performance. In her review, Sandi, (2013) adds that in tasks where the cognitive load is not excessive, mild stress tends to facilitate performance.

The effect of stress on task-switching specifically is also unclear. Goldfarb et al. (2017) found that cortisol (a stress hormone) response to stress enhanced participant’s ability to update task information they held in working memory, but reduced their performance when switching between different task demands. Similarly, Plessow et al. (2012) reported that stressed individuals took significantly longer to respond during task switches than task repetitions compared to controls. Liston et al. (2009) and Orem et al. (2008) also found that task-switching was impaired by chronic psychological stress. However, acute stress has also been reported to improve response time in task-switching trials without affecting accuracy (Lin et al., 2018).

### Hypotheses

The present study aims to clarify the relationship between stress and TWW. More specifically, we aimed to test whether stress is associated with TWW and whether it may also be a result of TWW. We also tested for the effects of stress on secondary task performance when TWW. Three hypotheses were formulated for the purposes of this study:

*Hypothesis 1*: High pre-task stress, whether psychological (H1a) or physiological (H1b), will lead to greater smartphone use during an unrestricted walking task.

This hypothesis is based on the literature suggesting that smartphones can be used to soothe the negative emotions caused by chronic or acute stressors (Billieux, 2012; Hunter et al., 2018).

*Hypothesis 2*: Smartphone use will decrease psychological stress (H2a) and simultaneously increase physiological stress (H2b) when TWW.

These hypotheses are based on the literature outlining the short-term effects of smartphone use on psychological stress (Hunter et al., 2018; Melumad and Pham, 2021) and on the literature examining the impact of technology use on physiological stress (Riedl, 2012; Afifi et al., 2018).

*Hypothesis 3*: There is a curvilinear relationship between physiological stress and TWW performance, wherein moderate physiological stress leads to better performance.

This hypothesis is based on Yerkes and Dodson (1908) inverted U-law, which suggests that cognitive performance is best at moderate stress levels. We make this hypothesis based on our biological motion perception task (explained hereafter), which generates little cognitive load. According to some researchers, moderate stress tends to enhance performance, particularly in these types of tasks (Sandi, 2013).

## Materials and methods

### Study design and participants

Participants performed two tasks in the same order so that the effects of phone use in Task A could be assessed during Task B. We measured psychological and physiological stress before and after each task. To control for circadian fluctuations in cortisol (Lupien et al., 2007), all tests were conducted between noon and 6:00 PM. Participants were recruited through our institution’s pool of participants and through social media. A total of 80 participants between the ages of 18 and 49 participated in the study. All participants received a compensation in the form of $50. This study was approved by our institution’s Ethics Research Committee under the certificate #2019–3,412 and was performed in accordance with the principles of the revised Helsinki Declaration of 2013. The data for this study is available at https://osf.io/82pvg.

### Procedure

The stimulus used for the spatial recognition task was a dynamic point-light representation of a walking human form as it is more easily adjusted to an optimal accuracy (i.e., approximately 80% accuracy in pilot testing) while also maintaining a high degree of ecological validity (Johansson, 1973). This point-light figure represents biological motion (BM) and is composed of 15 black dots which represent the head, shoulders, hips, elbows, wrists, knees, and ankles of the human figure (Thornton et al., 2002). Participants were instructed to verbally identify the walker’s direction (leftward or rightward) each time they heard the sound cue.

The figures were projected on the frontal screen; they were 1.08 meters in height and were presented walking forward, with a rightward or leftward deviation angle of 3.5° (or −3.5°) from the participant. A 1,000 ms auditory cue was delivered at 20s intervals and 500 ms before the presentation of each walking figure with a 50 ms jitter. Two speakers (Logitech, Switzerland) placed in front of the participants were used to deliver these cues. A stimulus response was considered correct when the participant accurately identified the direction in which the figure was headed (i.e., left or right).

The experimental procedure consisted of two main tasks and a practice task and lasted 50 min on average. Participants first performed a 3-min practice task during which they walked on the treadmill and responded to the visual stimuli. The practice task was meant to habituate participants to the treadmill speed and to the stimulus. Participants were asked to put their phone on “do not disturb” mode and to place it face-down on the console in front of them before this task. A total of sixwalker figures were presented during practice. If participants got more than 4 out of six responses wrong, or if they missed or did not respond to the stimulus, the practice task was repeated. During a repeated practice, the researcher gave the first three correct answers to the participant while being present in the room, and the participant was asked to respond to the last three trials. Once the practice task was over, participants were told they could remove the “do not disturb” mode on their phone. The participants’ personal phone remained on the front console for the duration of the next task.

The practice task was followed by two within-subject tasks which occurred in the same order for all participants: an unrestricted task (Task A) and a TWW task (Task B). Each task lasted 13 min and comprised 40 stimulus trials. During Task A, participants walked on the treadmill and responded to the walker stimulus. Before the task began, participants received the following information: (1) the task can be a little boring, (2) they should feel free to use their phone at any time if they wished, (3) using their phone was not dangerous or disturbing to the researchers, and (4) they should try nonetheless to respond correctly to the task and avoid taking or making calls. These instructions were devised to give participants free choice over whether and how long they used their personal phone during Task A. Unlike the other instructions given to participants, this information was provided verbally by the researcher in a spontaneous and familiar tone. Participants were not aware that their personal phone use would be monitored during this task. After Task A, participants who used their phone were asked to indicate *via* questionnaire the names of the applications they used and the estimated time they spent on each application.

After Task A, participants were instructed to store their personal phone in a locker outside of the room in preparation for the TWW Task (Task B). A smartphone was then provided to them. On average, there was a 5-min delay between Tasks A and B. During Task B, participants walked on the treadmill and responded to the walker figure stimulus. At the same time, they participated in a conversation with a confederate located in an adjacent room *via* text message. Conversations were led by the confederate through the use of a list of predefined topics. Topics were designed to be open-ended and to avoid strong emotional reactions (e.g., “What are your favorite television shows?,” “Where have you traveled lately?”). The confederate moved to a different conversation topic after 4–5 questions/answers on the same topic. The procedure used in Task B mirrors the one in Courtemanche et al. (2019).

**Figure 1:**
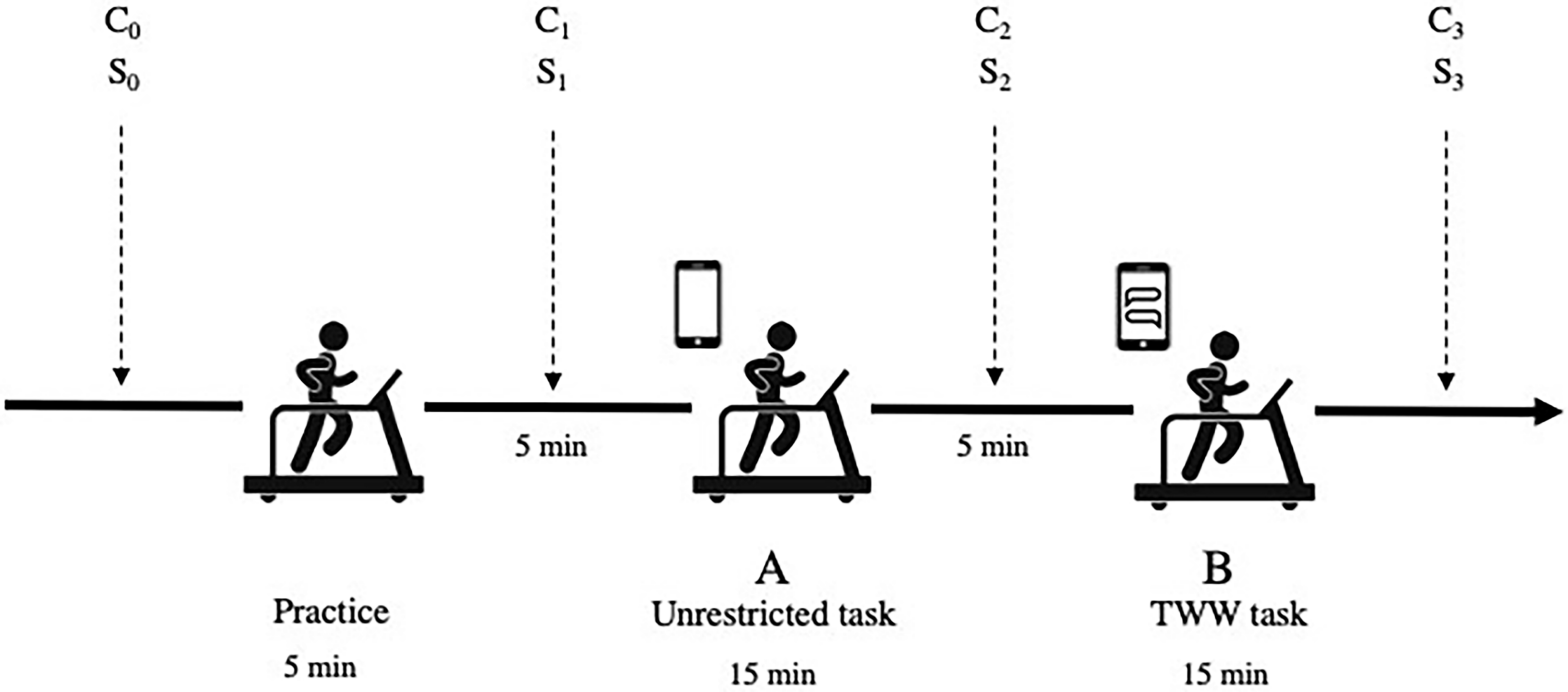
Test methodology.

Saliva samples were collected before and after each task, followed by a subjective stress measurement scale. To obtain a baseline stress measure for each participant, the first saliva sample and stress measure were taken upon arrival to the laboratory, before any instructions were given. A total of four paired saliva cortisol (C) and subjective stress (S) measures were thus obtained throughout the experiment: at baseline (C_0_ and S_0_), before Task A (C_1_ and S_1_), between Tasks A and B (C_2_ and S_2_), and after Task B (C_3_ and S_3_; see Figure 1).

### Apparatus

Our experiment reproduced the methodology used in the study of Courtemanche et al. (2019). The experiment took place in a room containing a treadmill (Tempo Fitness, Wisconsin, United States), a Marquee Ultra 8,500 projector with a resolution of 1,280 × 1,024 pixels, and a frontal projection screen. The treadmill speed was set to 0.8mph, a speed that was found to be the safest and most comfortable for performing the experiment during pilot testing. The treadmill was placed 2.44 m away from the projection screen. During the controlled TWW task (Task B), participants held a conversation *via* text message using a white iPhone 6S (Apple, United States). The conversation was carried out on Facebook Messenger, a popular messaging application.

### Measures

#### Psychological stress

Acute psychological stress levels were measured 4 times throughout the experiment: upon arrival to the laboratory (S_0_), before the unrestricted task (S_1_), between Tasks A and B (S_2_), and after Task B (S_3_; Figure 1). We measured acute psychological stress with an abridged version of the State Anxiety Inventory (Spielberger et al., 1983). This version contains six statements that participants rate on a four-point Likert scale ranging from 1 (“Not at all”) to 4 (“Very”). Statements include, “I feel calm,” “I feel tense,” and “I feel preoccupied.” Participants were instructed to respond according to how they felt in the present moment. This short version of the State Anxiety Inventory has been reported to yield comparable results to the longer version (Marteau and Bekker, 1992).

### Phone use

Phone use during Task A was measured first as a binary variable (i.e., “yes” or “no”). In addition, we measured the total number of phone uses during Task A. For the purposes of this study, a “phone use” was defined as any time a participant either (a) picked up their phone and looked at their screen, or (b) returned their eyes to their phone screen if they had already been holding it. Attentional shifts resulting from the presentation of a walker stimulus (i.e., when a participant looked away from her phone to give her response) were not counted as a separate phone use. Additionally, we measured the amount of time it took participants to use their phone for the first time (i.e., time to first use) and the total amount of time spent using their phone during the task. Following Task A, participants were asked to list the applications they had used and to estimate the amount of time in minutes they had spent on each. From this information, we retained the type of application on which the participant spent the most time (type of application used). The type of application used was coded according to the following categories: communication (e.g., texting, SMS, WhatsApp), social media (e.g., Facebook, Instagram, Snapchat), games, entertainment (e.g., Youtube, Netflix), school-or work-related (e.g., email), internet (e.g., browser), or other. Phone use (yes/no), number of uses, total time, time to first use, and type of application used were all the phone-use variables included in our statistical analysis.

### Physiological stress

Saliva samples were collected 4 times throughout the experiment: upon arrival to the laboratory (C_0_), before Task A (C_1_), between Tasks A and B (C_2_), and after Task B (C_3_; Figure 1). Each sample was approximately 2 ml. Frozen samples were brought to room temperature and centrifuged at 1,500×g (3,000 rpm) for 15 min. High-sensitivity enzyme immunoassays were used (Salimetrics^®^, No. 1–3,102, sensitivity: 0.012–3 μg/dl). Inter-assay and intra-assay coefficients of variance were below 4.69%. All assays were duplicated and averaged.

### Statistical analysis

Statistical analyses were performed using SAS 9.4 for Windows. Sex and age were included in every model as covariates. In addition, given the presence of sex-specific responses in physiological responses to stress (Kudielka and Kirschbaum, 2005), additional separate analyses were performed for women and for men, and *p*-values were corrected using the Bonferroni correction in these instances.

Cortisol and psychological stress measures were adjusted for baseline in the following way:


$$\delta C_{m}^{p}=C_{m}^{p}-C_{0}^{p}$$

Where p refers to a given participant, *C_m_* refers to a given cortisol measure taken after baseline (i.e., C_1_, C_2_, or C_3_), and C_0_ refers to cortisol measured at baseline.

Additionally, we calculated cortisol changes during tasks (i.e., delta cortisol) in the following way:


$$\Delta C_{m,n}^{p}=C_{m}^{p}-C_{n}^{p}$$

Where p refers to a given participant, $C_{m}$ refers to a given cortisol measure taken after a task (i.e., C_2_, or C_3_), and *C_n_* refers to a given cortisol measure taken before that task (i.e., C_1_ or C_2_).The same manipulations were performed on the psychological stress measures collected *via* questionnaire in order to obtain δS and ΔS.

The following analyses were performed for each hypothesis: for H1, we used regression models appropriate for the type of data and the normality of the distributions to verify whether psychological (H1a) or physiological (H1b) stress was associated with any of the phone use variables (yes/no) (logistic), number of uses (Poisson), total time (linear) and time to first use (exponential). For H2, we used the same regression models to verify whether the phone use variables measured in Task A predicted psychological (H2a) and/or physiological (H2b) stress after Task A. For H3, we used the same regression models with a quadratic term to verify whether there was a quadratic relationship between performance in Task B and stress measured during or after Task A. All results were adjusted for multiple comparisons.

## Results

### Sample and descriptive statistics

A total of 80 people participated in the study, including 44 (55%) women and 36 (45%) men. Demographics and a breakdown of main task variables are presented in Table 1. The mean age was 23.6 years (SD = 5.34). A total of 53 participants (66%) used their personal phone during Task A. Average usage time was 4.4 (SD = 4.65) minutes and average time to first use was 2.9 min (SD = 3.52). The average number of phone uses throughout the task was 3.36 (SD = 4.31). The top three types of application most used were social media (*n* = 28, 52%), communication (*n* = 13, 24%) and games, and other (*n* = 4, 7%). On average, participants responded correctly to 82% of the trials in Task A and to 81% of the trials in Task B, which is in line with pilot testing and previous results from Courtemanche et al. (2019).

**Table 1:** Demographics and task variables by sex.

	Males	Females	Total
*n*	36	44	80
Average age	25.13 (SD = 6.26)	22.39 (SD = 4.10)	23.64 (SD = 5.34)
**Task A**			
Accuracy (% correct)	86.57 (SD = 13.18)	78.22 (SD = 16.14)	82.00 (SD = 15.35)
Used phone (yes)	27	26	53
Total time of use (avg. mins.)	5.03 (SD = 4.68)	3.90 (SD = 4.63)	4.42 (SD = 4.65)
Time to first use (avg. mins.)	2.39 (SD = 3.05)	3.51 (SD = 3.99)	2.93 (SD = 3.52)
Number of phone uses (avg.)	3.97 (SD = 3.96)	2.86 (SD = 4.57)	3.36 (SD = 4.31)
Type of app most used	Social media	Social media	Social media
**Task B**			
Accuracy (% correct)	84.15 (SD = 14.00)	79.87 (SD = 14.66)	81.81 (SD = 14.46)
**Average cortisol (μg/dl, SD)**			
C_0_	0.19 (0.12)	0.20 (0.17)	0.19 (0.14)
C_1_	0.17 (0.10)	0.18 (0.17)	0.17 (0.14)
C_2_	0.15 (0.08)	0.14 (0.11)	0.14 (0.10)
C_3_	0.12 (0.06)	0.11 (0.06)	0.12 (0.06)
**Average state anxiety inventory score (SD)**			
S_0_	30.17 (8.05)	33.12 (7.96)	31.67 (8.34)
S_1_	29.67 (6.44)	32.81 (7.22)	31.25 (7.49)
S_2_	31.58 (7.53)	32.47 (8.13)	31.96 (7.90)
S_3_	30.02 (6.73)	31.81 (7.32)	30.92 (7.41)

### Relationship between stress and phone use (H1)

**Figure 2:**
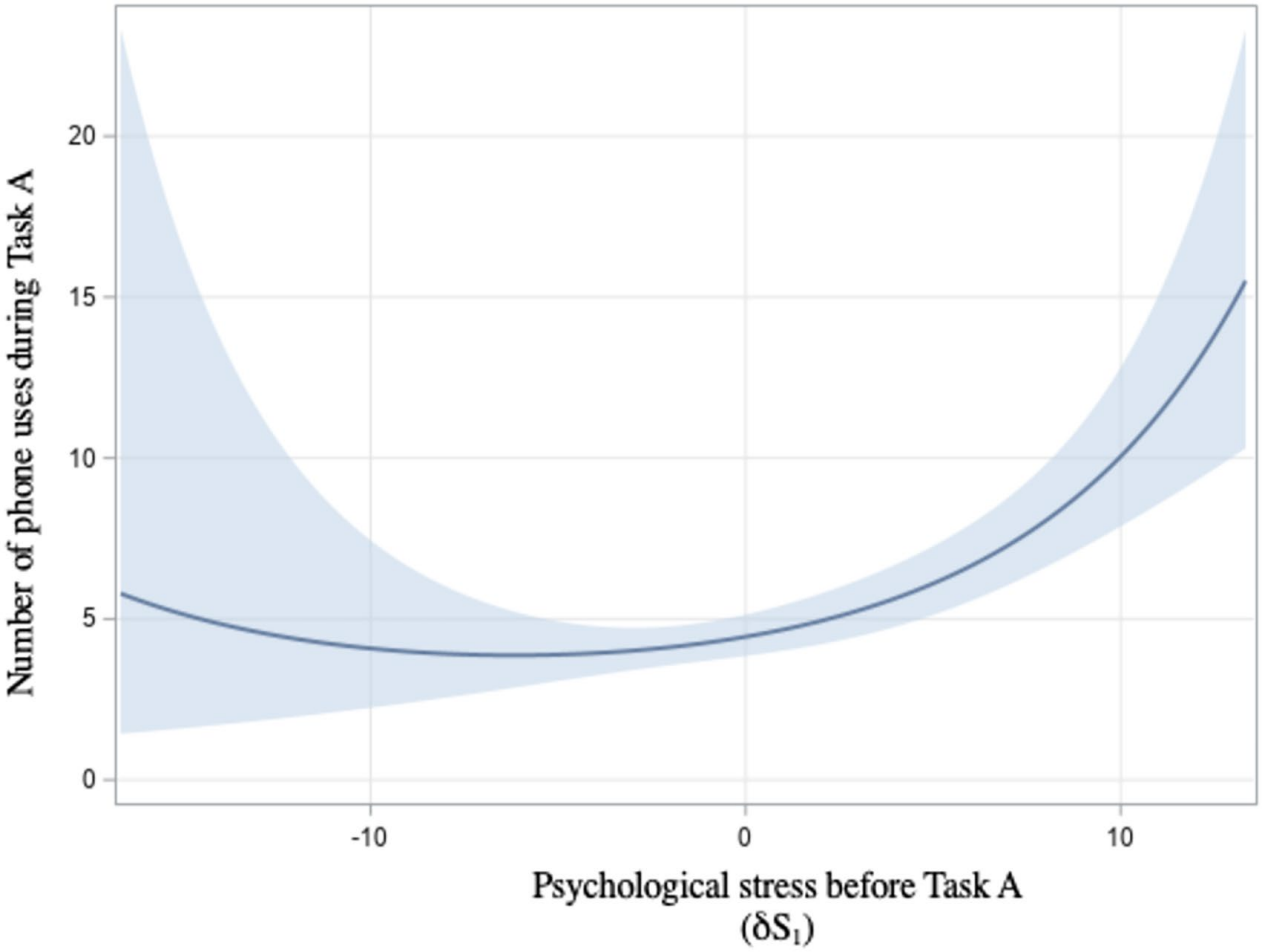
Relationship between psychological stress before Task A and number of phone uses with 95% confidence interval.

Our first hypothesis was concerned with associating stress and texting while walking. We expected that stress, either physiological (H1a) or psychological (H1b), would correlate with smartphone use in Task A. The dependent variables included in the analysis were the phone use variables measured during Task A (phone use, total time of use, number of uses, and time to first use). The complete statistical results can be seen in the Supplementary Material for both H1 and H2. Baseline-adjusted psychological stress before Task A (δS_1_) was associated with a greater number of phone uses [t(50) = 5.29, *p* < 0.0001; Figure 2]. Sex was not significantly associated with phone uses in this analysis [t(50) = 1.13, *p* = 0.26].

**Figure 3:**
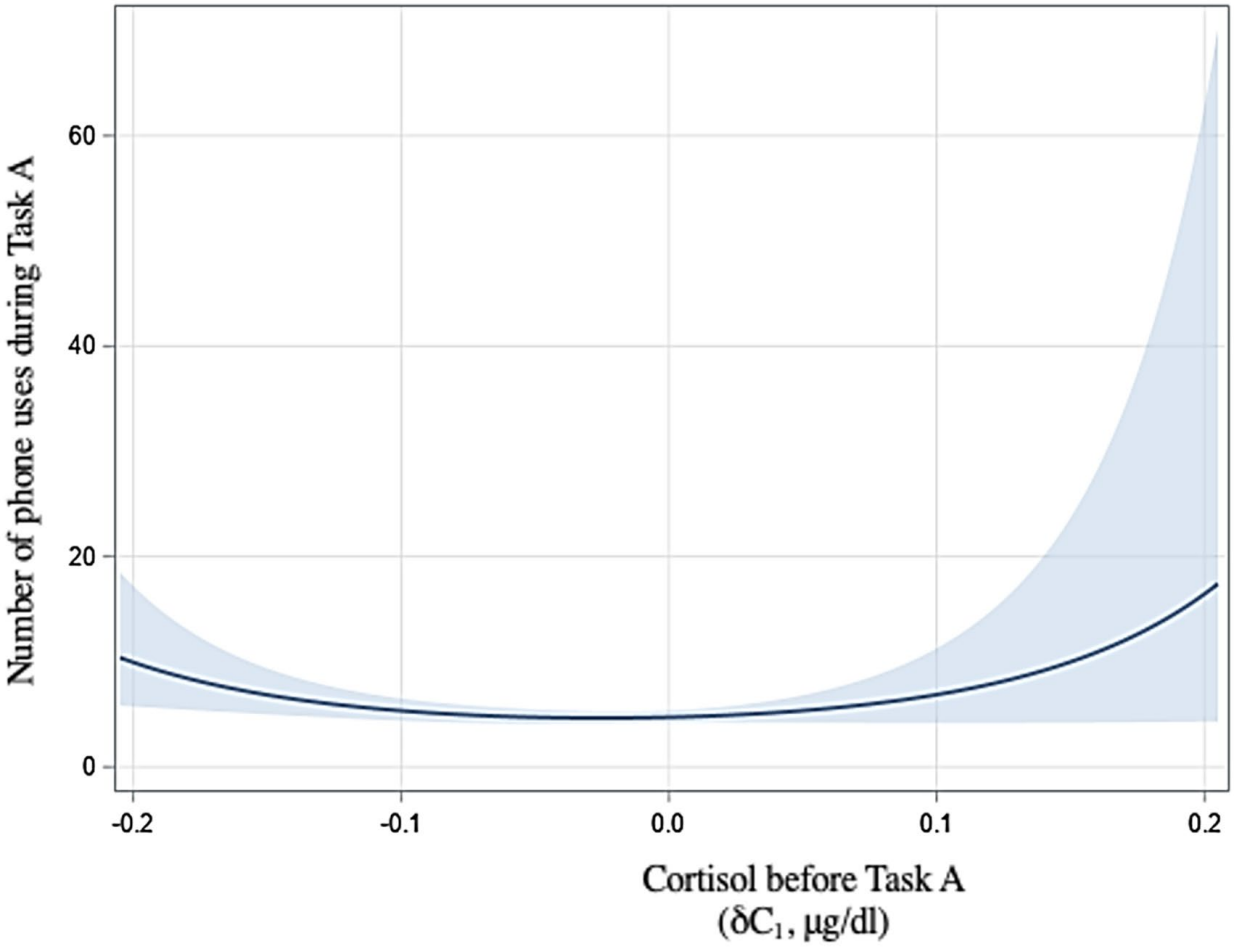
Relationship between physiological stress before Task A and number of phone uses with 95% confidence interval.

In addition, baseline-adjusted cortisol before Task A (δC_1_) had a quadratic effect on the number of times participants used their phones during the task [t(49) = 2.09, *p* = 0.04; Figure 3], but not on other phone use variables. Sex was not significantly associated with phone uses in this analysis [t(49) = 0.39, *p* = 0.70].

In light of these analyses, we find that H1 is supported. Higher psychological stress before Task A was associated with a greater number of phone uses during the task (H1a) and a quadratic relationship was found between baseline-adjusted physiological stress before Task A and the number of phone uses (H1b).

### Effects of phone use on stress (H2)

Our second hypothesis tested the effects of using one’s phone during Task A on psychological and physiological stress. We expected that using one’s phone during Task A would be associated with a reduction of psychological stress (H2a) and, simultaneously, an increase of physiological stress (H2b).

**Figure 4:**
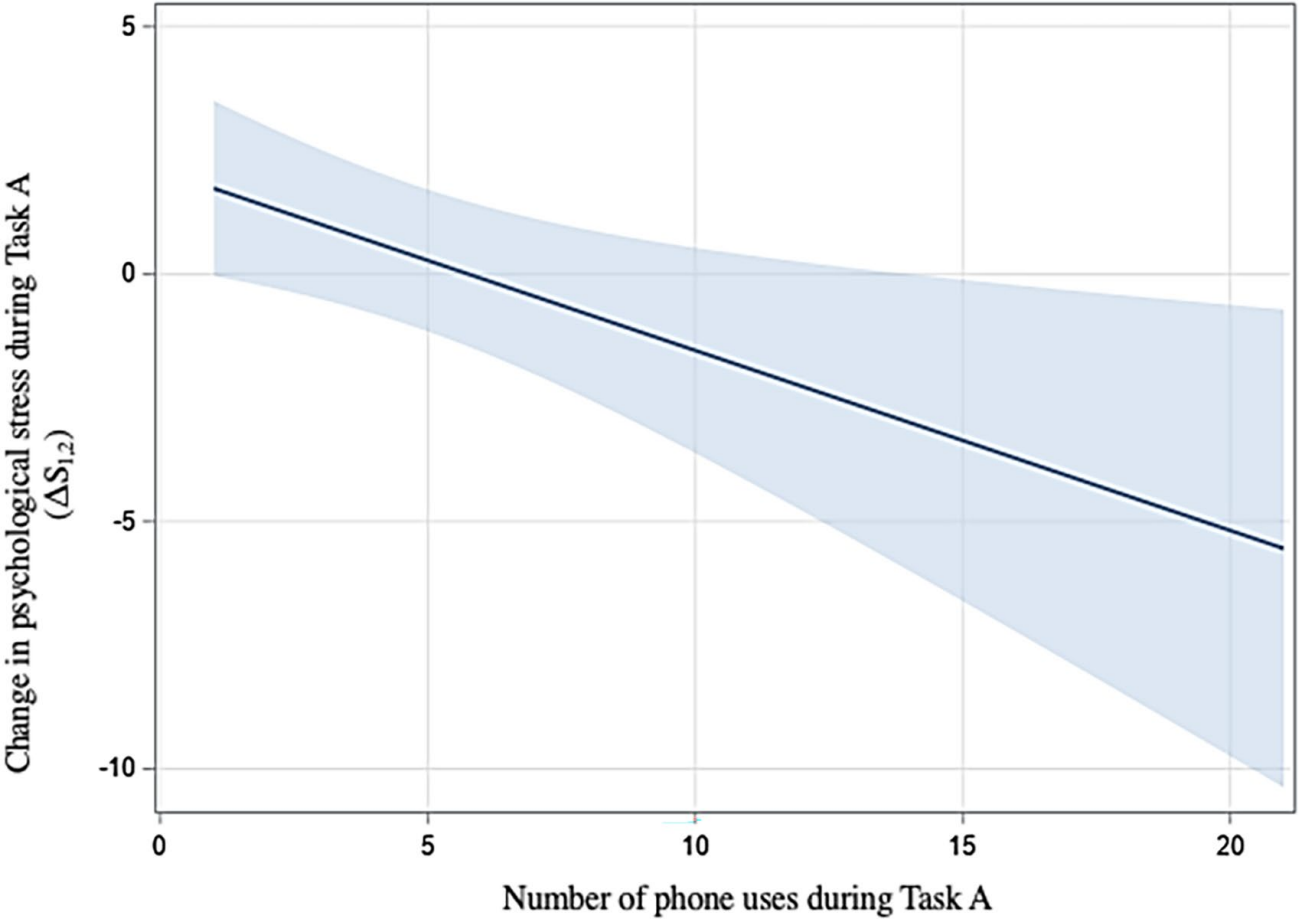
Relationship between number of phone uses and psychological stress (H2a) with 95% confidence interval.

In regard to H2a, phone use (yes/no), time to first use, and total time of use were not significantly related to psychological stress after Task A. However, there was a significant negative relationship between number of phone uses and changes in psychological stress throughout the task (ΔS_1,2_); this relationship was only significant in women [t(24) = −2.42, *p* = 0.023, Figure 4].

**Figure 5:**
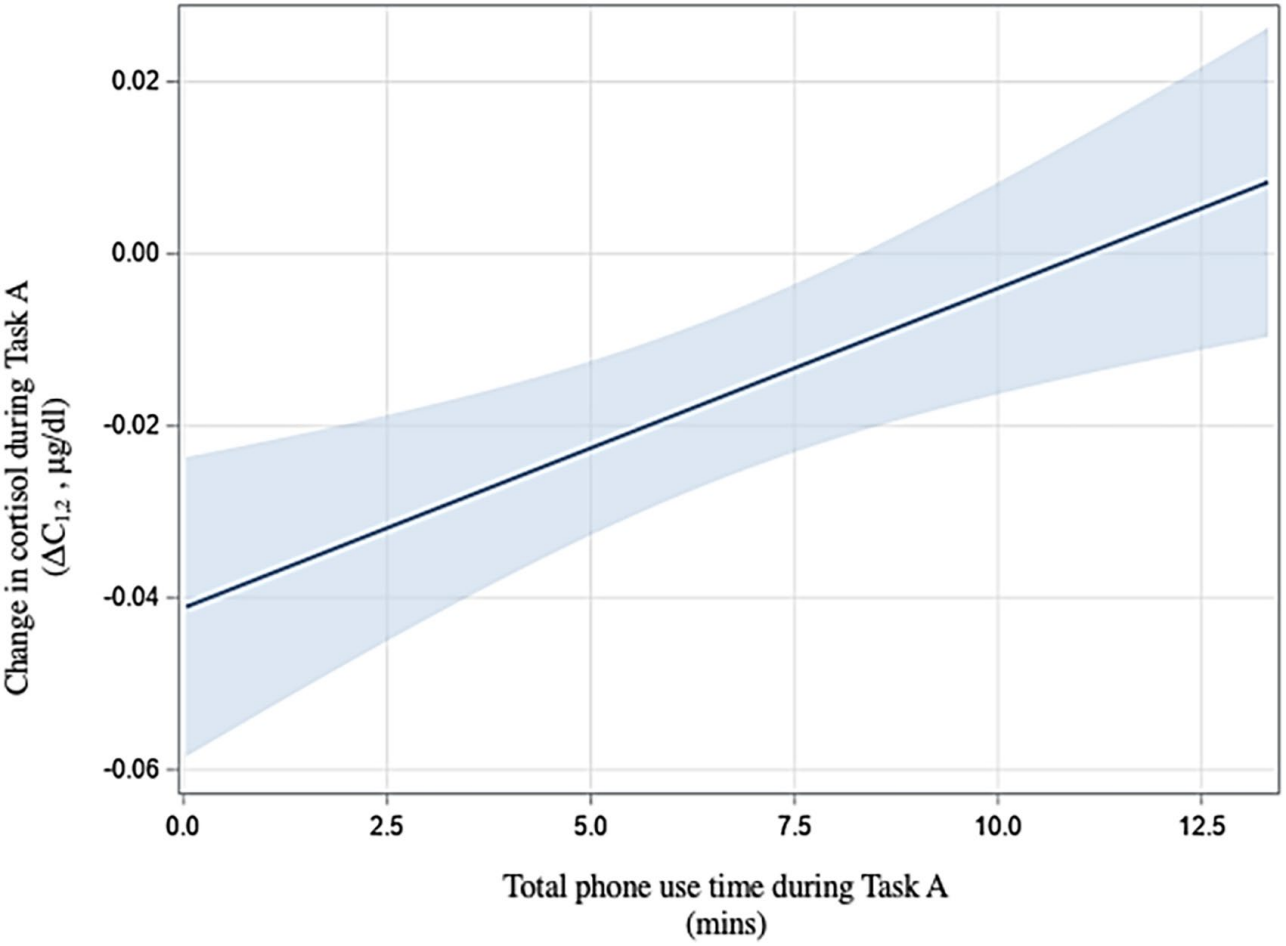
Relationship between phone use time and physiological stress (H2b) with 95% confidence interval.

Regarding H2b, we found no relationship between phone usage (yes/no), time to first use, or number of uses and physiological stress during Task A. We found that longer phone use (in minutes) during Task A was associated with a greater increases in cortisol during the task [ΔC_1,2_; t(50) = 3.17, *p* = 0.003; Figure 5]. Sex did not have a significant effect on cortisol change [t(50) = −0.47, *p* = 0.64] in this analysis.

We thus found H2 to be partially supported: a higher number of phone uses did decrease psychological stress during Task A in women (H2a). Additionally, longer phone use during Task A was associated with a greater increases in physiological stress during the task for all participants (H2b).

### Effects of cortisol on performance (H3)

**Figure 6:**
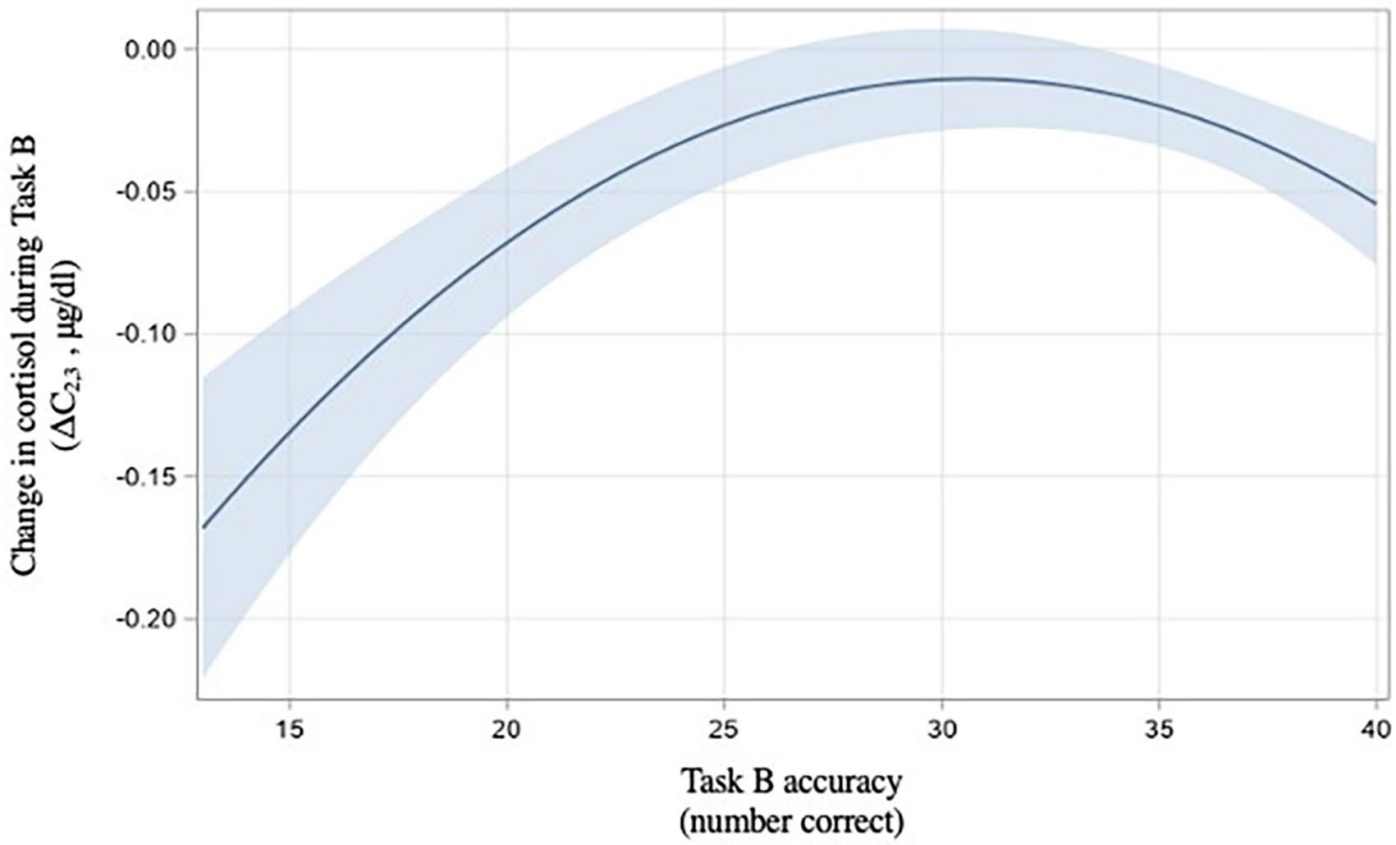
Relationship between Task B performance and cortisol (H3) with 95% confidence intervals with 95% confidence interval.

Our third hypothesis was concerned with the relationship between cortisol and performance. We hypothesized that there would be a curvilinear relationship between cortisol and performance in Task B. Using changes in cortisol during the task (ΔC_2,3_), we found a curvilinear relationship between cortisol change and task accuracy [t(75) = −2.83, *p* = 0.006, Figure 6].

We therefore find H3 to be supported: moderate performance was associated with higher cortisol changes during the task.

## Discussion

The purpose of the present study was to examine the relationship between psychological and physiological stress and smartphone use in the context of texting while walking (TWW).

Our first objective was to examine stress as a potential antecedent of TWW in the unrestricted task (Task A), where participants were free to use their phone if they wished. We found that perceived stress before the unrestricted task was associated with a greater number of phone uses (H1a). Thus, participants who experienced an increase in psychological stress before the unrestricted task relative to baseline were more likely to use their phones several times during this task. This finding is congruent with the literature suggesting that psychological stress can be a precursor to smartphone use (Boumosleh and Jaalouk, 2017; Cho et al., 2017; Kuang-Tsan and Fu-Yuan, 2017). More specifically, some researchers suggest that smartphones can serve as a strategy to reduce psychological stress (Hunter et al., 2018). The result showing that participants who experienced increases in psychological stress proceeded to use their phones several times seems congruent with this view. Interestingly, this result is specific to the number of phone uses, and not to the total time of use. It is therefore possible that increases in psychological stress are associated with a greater tendency or need to check one’s phone in a compulsive manner, rather than the tendency to use it longer.

Changes in physiological stress, as well, were found to be a determinant of smartphone use. We found that cortisol levels before the unrestricted task had a curvilinear effect on the number of phone uses during the task (H1b). This suggests that those participants who experienced either a drop or an increase in cortisol relative to baseline before the task were most likely to use their phones several times. To our knowledge, no literature has yet studied physiological stress as a potential predictor of smartphone use. However, these results suggest that continuous or impulsive smartphone checking behavior could be associated with cortisol fluctuation. It is possible that phone checking behavior in those for whom cortisol decreased was due to boredom or to a need for stimulation, and in those from whom cortisol increased, to a need for distraction in response to stress.

Our second objective was to investigate the effects of TWW on psychological and physiological stress. To achieve this, stress levels after the unrestricted task were compared between participants who used their phones extensively and those who used it less. We found that a greater number of phone uses was associated with lower psychological stress, through this relationship was only significant in women (H2a). Additionally, longer phone use (in minutes) during the unrestricted task was associated with greater increases in cortisol (H2b) during the task. Cortisol therefore tended to increase as the time spent using one’s phone increased. Additionally, in women, a higher number of phone uses was related to a decrease in psychological stress during the task as reporter in other studies (Misigo, 2015; Xu et al., 2015; Anbumalr et al., 2017). This is a similar effect to that of Hunter et al. (2018) social blanket theory, and observed in the publication of Melumad and Pham (2021): the availability of the participant’s phone, and in this case, more phone checking behavior (i.e., phone uses) seemed to reduce psychological stress during the task. While the research of Hunter et al. (2018) found no effect of smartphone use on salivary cortisol, Riedl et al. (2012) found that the use of technology tends to be associated with an increase in salivary cortisol. As previously mentioned, measures of psychological and physiological measures of stress do not always correlate (Hjortskov et al., 2004; Campbell and Ehlert, 2011; Ali et al., 2017). It is possible that the scale employed, the short form of the State Anxiety Inventory (SAI) did not capture the overall stress response experienced by the participants. Hjortskov et al. (2004) mention that different scales will measure responses to different types of mental stressors. It is possible that the response measured by the SAI was diminished when holding the smartphone, as proposed by Hunter’s theory, while the overall stress response remained and was captured in the physiological measurement. Further research will be needed to truly understand why this difference between psychological and physiological measures appears.

Our third objective was to evaluate the relationship between physiological stress associated with smartphone use and performance in the TWW secondary task (i.e., Task B; H3). Analyses revealed that performance in the TWW task had a curvilinear effect on cortisol levels throughout the task (i.e., delta cortisol). This suggests that during the TWW task, cortisol tended to decrease the most for those participants who performed very well or very poorly. Those who performed moderately well experienced the highest levels of cortisol variation. These participants were likely making a conscious effort to respond to the perception task, while the high performers likely found the task too easy, and the low performers, too hard, leading to abandonment. It is important to note that participants did not receive feedback during the task and were therefore unaware of their general performance. However, we believe that an inconsistent pattern of response (i.e., moderate performance) may reflect conscious effort, whereas consistent performance, be it correct or incorrect, likely indicates that participants did not experience an adequate dual-task challenge. It is possible that participants who exerted a conscious effort to perform throughout the task experienced higher levels of cortisol as a result.

Through our results we found confirmatory evidence for the theory advanced by Billieux (2012) and Hunter et al. (2018) whereby smartphone use can act as a coping mechanism for stress. Our study revealed that higher psychological stress before the unrestricted task led to a greater number of phone uses. This suggests that phone use, in particular repeated phone-checking behavior, could be a response to perceived stress. In addition, our study is the first, to our knowledge, to directly examine the effect of cortisol levels on subsequent phone use. In this regard, we found that changes in cortisol relative to baseline (i.e., either a decrease or increase in cortisol) were associated with more phone uses during the unrestricted task.

The present study also helps to clarify the effect that phone multitasking can have on stress. We found evidence for the theory proposed by Hunter et al. (2018) whereby phones (in this case, not only their presence, but actual use) can help reduce psychological stress. In our study, a greater number of phone uses is related to lower psychological stress, although this relationship was only significant in women. Moreover, this finding and the above corroborate the results of Cougle et al. (2011) and Nikmanesh et al. (2014) implying that there is a cyclical relationship between stress and smartphone use. Smartphone application developers could use this information to introduce system warnings related to smartphone use and stress. For instance, users could be notified and made aware of the dangers associated with frequent phone-checking behavior and its relationship with stress. In addition, policy makers intending to reduce TWW may use this information to caution pedestrians on the effects that stress can have on both their smartphone use and their security.

Finally, the present study also examined the effect of physiological stress on performance in a texting-while-walking task. In this regard, we found confirmatory evidence for Yerkes and Dodson (1908) inverted U-law, whereby peak cortisol levels are associated with moderate performance. This likely implies that making a conscious effort to perform well (in this case, to successfully identify and avoid obstacles during distracted walking) can lead to a moderate increase in cortisol. Thus, making a conscious effort to multitask can be physiologically stressful.

The results of this study should be interpreted in view of its limitations. Firstly, while our study offers a first exploration of the relationship between stress and phone use, it did not manipulate stress in participants. In order to confirm a causal relationship between stress and phone use, therefore, further studies will need to be conducted where stress is also manipulated (for example, through the use of the Trier Social Stress Test (Kirschbaum et al., 1993). A study in which stress is more severe may show stronger results. In addition, we did not measure reaction time during the TWW task (i.e., time between stimulus onset and response), which prevented us from examining the effects of stress on this aspect of cognitive performance. Finally, we acknowledge that salivary cortisol may be affected by physical activity (Gatti and De Palo, 2011), however the walking speed in this study was so slow that we do not believe it had an effect. Indeed, slow walking on a treadmill has been shown to have no significant impact on salivary cortisol (Schmidt-Kassow et al., 2014).

## Conclusion

Smartphones have become ubiquitous in today’s society, the multitasking possibilities they provide have drastically changed our lifestyles for the better or the worse. As stress is a fundamental psychological and physiological part of life, understanding the relationship between smartphones and stress, especially in the context of one of the most common types of multitasking, should lead to better technology and better practices of personal and societal technology management. The current study adds to the knowledge on the interrelations between smartphone use and stress. We confirmed the social blanket theory (Hunter et al., 2018), showing that individuals experiencing more stress will be more likely to use their smartphones given the opportunity. We also see that more use does indeed reduce the psychological stress perceived by the women in our sample, however longer use of the smartphone leads to an increase in physiological stress. Finally, we find a curvilinear relationship between cortisol and secondary task performance, suggesting participants experiencing a moderate level of challenge experienced higher levels of stress. This could be indicative of the stress experienced in everyday experiences of texting while walking and should be taken into consideration when working to improve pedestrian safety.

## Data availability statement

The datasets presented in this study can be found in online repositories. The names of the repository/repositories and accession number(s) can be found at: https://osf.io/82pvg.

## Ethics statement

The studies involving human participants were reviewed and approved by Research Ethics Board (REB) of HEC Montreal. The patients/participants provided their written informed consent to participate in this study.

## Author contributions

All authors have participated in the design of the research and revision of the manuscript. P-ML, SS, and SL supervised the data acquisition. MA conducted the data collection and wrote the initial draft with ÉL-L. ÉL-L wrote the final manuscript. All authors contributed to the article and approved the submitted version.

## Funding

This work was supported by the Social Sciences and Humanities Research Council of Canada grant number 435-2020-1303.

## Conflict of interest

The authors declare that the research was conducted in the absence of any commercial or financial relationships that could be construed as a potential conflict of interest.

## Publisher’s note

All claims expressed in this article are solely those of the authors and do not necessarily represent those of their affiliated organizations, or those of the publisher, the editors and the reviewers. Any product that may be evaluated in this article, or claim that may be made by its manufacturer, is not guaranteed or endorsed by the publisher.
