# Supplementary material for: Stress can lead to an increase in smartphone use in the context of texting while walking
Source: Front Psychol. 2022 Sep 8;13:760107. doi: 10.3389/fpsyg.2022.760107 (PMC9496168; doi:10.3389/fpsyg.2022.760107)
Supplement: Supplementary file 1 [file Data_Sheet_1.PDF]

Hypothesis	Model	Effect	Estimate	SE	DF	t Value	p	95% CI	
								LL	UL
H1	Task A number of phone uses	Intercept	1.509	0.097	50	15.61	**<0.001**	1.315	1.704
		Cortisol before Task A	-1.343	1.207	50	-1.11	0.271	-3.766	1.081
		Sex	0.119	0.124	50	0.97	0.339	-0.129	0.367
		Intercept	1.483	0.093	50	15.96	**<0.001**	1.296	1.670
		Psychological stress before task A	0.068	0.013	50	5.29	**<0.001**	0.042	0.094
		Sex	0.140	0.124	50	1.13	0.262	-0.108	0.388
		Intercept	1.527	0.096	49	15.92	**<0.001**	1.334	1.720
		Cortisol before Task A	1.114	1.585	49	0.7	0.485	-2.071	4.300
		Quadratic Cortisol before Task A	23.577	11.272	49	2.09	**0.042**	0.925	46.228
		Sex	0.050	0.129	49	0.39	0.699	-0.210	0.310
		Intercept	1.431	0.097	49	14.73	**<0.001**	1.236	1.626
		Psychological stress before task A	0.046	0.017	49	2.74	**0.009**	0.012	0.080
		Quadratic Psychological stress before task A	0.003	0.002	49	1.88	0.066	0.000	0.007
		Sex	0.124	0.124	49	1	0.323	-0.125	0.373
	Task A phone use	Intercept	0.320	0.318	77	1.01	0.318	-0.314	0.953
		Cortisol before Task A	-2.490	4.383	77	-0.57	0.572	-11.218	6.238
		Sex	0.727	0.493	77	1.47	0.144	-0.255	1.709
		Intercept	0.394	0.311	77	1.27	0.210	-0.226	1.014
		Psychological stress before task A	0.058	0.048	77	1.21	0.232	-0.038	0.153
		Sex	0.755	0.497	77	1.52	0.133	-0.235	1.745
		Intercept	0.405	0.333	76	1.22	0.227	-0.258	1.068
		Cortisol before Task A	-3.685	4.784	76	-0.77	0.444	-13.214	5.844
		Quadratic Cortisol before Task A	-30.199	32.054	76	-0.94	0.349	-94.040	33.642
		Sex	0.714	0.496	76	1.44	0.154	-0.273	1.701
		Intercept	0.692	0.358	76	1.93	0.057	-0.021	1.406
		Psychological stress before task A	0.051	0.050	76	1.03	0.308	-0.048	0.150
		Quadratic Psychological stress before task A	-0.011	0.006	76	-1.78	0.079	-0.023	0.001
		Sex	0.713	0.513	76	1.39	0.169	-0.308	1.735

		Intercept	6.925	0.886	50	7.81	**<0.001**	5.145	8.705
		Cortisol before Task A	22.725	11.923	50	1.91	0.062	-1.223	46.674
		Sex	0.271	1.162	50	0.23	0.817	-2.063	2.605
		Intercept	6.352	0.853	50	7.45	**<0.001**	4.640	8.065
		Psychological stress before task A	-0.114	0.140	50	-0.81	0.421	-0.394	0.168
		Sex	0.349	1.195	50	0.29	0.771	-2.051	2.749
		Intercept	6.911	0.897	49	7.7	**<0.001**	5.108	8.714
	Task A total time of phone use	Cortisol before Task A	20.371	16.868	49	1.21	0.233	-13.527	54.268
		Quadratic Cortisol before Task A	-25.037	125.630	49	-0.2	0.843	######	227.420
		Sex	0.332	1.213	49	0.27	0.785	-2.106	2.770
		Intercept	6.131	0.939	49	6.53	**<0.001**	4.243	8.019
		Psychological stress before task A	-0.153	0.156	49	-0.98	0.333	-0.467	0.161
		Quadratic Psychological stress before task A	0.012	0.021	49	0.58	0.564	-0.030	0.055
		Sex	0.348	1.203	49	0.29	0.774	-2.069	2.765
H1		Intercept	1.226	0.208	49	5.89	**<0.001**	0.808	1.644
		Cortisol before Task A	-1.485	2.962	49	-0.5	0.618	-7.438	4.467
		Sex	-0.399	0.279	49	-1.43	0.159	-0.959	0.161
		Intercept	1.258	0.200	49	6.28	**<0.001**	0.855	1.660
		Psychological stress before task A	0.007	0.034	49	0.2	0.839	-0.061	0.075
		Sex	-0.388	0.279	49	-1.39	0.170	-0.948	0.172
		Intercept	1.223	0.208	48	5.89	**<0.001**	0.805	1.640
	Task A time to first phone use	Cortisol before Task A	-2.229	3.679	48	-0.61	0.548	-9.626	5.169
		Quadratic Cortisol before Task A	-8.686	27.642	48	-0.31	0.755	-64.263	46.891
		Sex	-0.378	0.288	48	-1.31	0.196	-0.956	0.201
		Intercept	1.296	0.215	48	6.02	**<0.001**	0.863	1.729
		Psychological stress before task A	0.014	0.036	48	0.4	0.690	-0.058	0.087
		Quadratic Psychological stress before task A	-0.002	0.005	48	-0.51	0.615	-0.012	0.007
		Sex	-0.378	0.280	48	-1.35	0.184	-0.941	0.186

		Intercept	-0.826	0.954	77	-0.87	0.389	-2.724	1.073
		Task A phone use	0.885	1.105	77	0.8	0.426	-1.316	3.085
		Sex	2.107	1.050	77	2.01	**0.048**	0.015	4.198
		Intercept	1.707	1.139	50	1.5	0.140	-0.581	3.994
		Task A number of phone uses	-0.282	0.145	50	-1.95	0.057	-0.572	0.009
	Psychological stress after Task A, both genders	Sex	1.627	1.284	50	1.27	0.211	-0.952	4.206
		Intercept	1.129	1.363	50	0.83	0.412	-1.609	3.867
		Task A total time of phone use	-0.118	0.156	50	-0.76	0.453	-0.430	0.195
		Sex	1.513	1.323	50	1.14	0.258	-1.144	4.171
		Intercept	-0.653	1.170	49	-0.56	0.580	-3.005	1.699
		Task A time to first phone use	0.262	0.192	49	1.36	0.180	-0.125	0.649
		Sex	1.878	1.344	49	1.4	0.169	-0.822	4.578
		Intercept	-0.053	0.012	77	-4.47	**<0.001**	-0.077	-0.029
		Task A phone use	0.031	0.014	77	2.28	**0.026**	0.004	0.059
		Sex	0.009	0.013	77	0.71	0.478	-0.017	0.035
		Intercept	-0.015	0.010	50	-1.55	0.127	-0.035	0.004
H2		Task A number of phone uses	0.000	0.001	50	-0.03	0.979	-0.003	0.002
	Cortisol after Task A, both genders	Sex	-0.003	0.011	50	-0.29	0.773	-0.025	0.019
		Intercept	-0.039	0.010	50	-3.77	**<0.001**	-0.060	-0.018
		Task A total time of phone use	0.004	0.001	50	3.17	**0.003**	0.001	0.006
		Sex	-0.005	0.010	50	-0.47	0.643	-0.025	0.015
		Intercept	-0.014	0.010	49	-1.37	0.178	-0.033	0.006
		Task A time to first phone use	0.000	0.002	49	-0.3	0.767	-0.004	0.003
		Sex	-0.004	0.011	49	-0.34	0.735	-0.027	0.019
		Intercept	-1.296	1.035	42	-1.25	0.218	-3.386	0.793
		Task A phone use	1.681	1.347	42	1.25	0.219	-1.037	4.399
		Intercept	2.092	1.039	24	2.01	0.055	-0.052	4.236
		Task A number of phone uses	-0.364	0.150	24	-2.42	**0.023**	-0.674	-0.054
	Psychological stress after Task A, women	Intercept	1.409	1.473	24	0.96	0.348	-1.631	4.449
		Task A total time of phone use	-0.162	0.192	24	-0.85	0.406	-0.557	0.233
		Intercept	-0.628	1.146	23	-0.55	0.589	-2.998	1.742
		Task A time to first phone use	0.255	0.218	23	1.17	0.254	-0.196	0.706

		Intercept	-0.062	0.016	42	-4.01	**<0.001**	-0.093	-0.031
		Task A phone use	0.047	0.020	42	2.32	**0.025**	0.006	0.088
		Intercept	-0.018	0.007	24	-2.4	**0.025**	-0.033	-0.002
	Cortisol after task A, women	Task A number of phone uses	0.000	0.001	24	0.46	0.649	-0.002	0.003
		Intercept	-0.033	0.008	24	-3.83	**0.001**	-0.050	-0.015
		Task A total time of phone use	0.003	0.001	24	2.46	**0.021**	0.000	0.005
		Intercept	-0.011	0.007	23	-1.48	0.153	-0.026	0.004
		Task A time to first phone use	-0.001	0.001	23	-0.84	0.410	-0.004	0.002
		Intercept	2.222	1.624	34	1.37	0.180	-1.079	5.523
		Task A phone use	-0.370	1.876	34	-0.2	0.845	-4.182	3.441
		Intercept	2.555	1.782	25	1.43	0.164	-1.116	6.226
H2	Psychological stress after Task A, men	Task A number of phone uses	-0.134	0.277	25	-0.48	0.634	-0.704	0.437
		Intercept	2.319	1.970	25	1.18	0.250	-1.739	6.377
		Task A total time of phone use	-0.070	0.250	25	-0.28	0.783	-0.585	0.446
		Intercept	1.198	1.313	25	0.91	0.371	-1.507	3.902
		Task A time to first phone use	0.273	0.346	25	0.79	0.437	-0.439	0.986
		Intercept	-0.025	0.014	34	-1.75	0.090	-0.055	0.004
		Task A phone use	0.007	0.017	34	0.4	0.690	-0.027	0.041
		Intercept	-0.013	0.017	25	-0.81	0.426	-0.047	0.021
	Cortisol after Task A, men	Task A number of phone uses	-0.001	0.003	25	-0.38	0.707	-0.006	0.004
		Intercept	-0.051	0.017	25	-3.08	**0.005**	-0.085	-0.017
		Task A total time of phone use	0.005	0.002	25	2.3	**0.030**	0.001	0.009
		Intercept	-0.020	0.012	25	-1.64	0.114	-0.045	0.005
		Task A time to first phone use	0.001	0.003	25	0.21	0.838	-0.006	0.007
